# Fortification of *Acidophilus*-*bifidus*-*thermophilus* (ABT) Fermented Milk with Heat-Treated Industrial Yeast Enhances Its Selected Properties

**DOI:** 10.3390/molecules26133876

**Published:** 2021-06-25

**Authors:** Fouad M. F. Elshaghabee, Ahmed A. Abd El-Maksoud, Sulaiman Ali Alharbi, Saleh Alfarraj, Mahmoud S. M. Mohamed

**Affiliations:** 1 Dairy Science Department, Faculty of Agriculture, Cairo University, Giza 12613, Egypt; ahmed_ali@cu.edu.eg; 2 Department of Botany and Microbiology, College of Science, King Saud University, P.O. Box 2455, Riyadh 11451, Saudi Arabia; sharbi@ksu.edu.sa; 3 Zoology Department, College of Science, King Saud University, Riyadh 11451, Saudi Arabia; salfarraj@hotmail.com; 4 Department of Botany and Microbiology, Faculty of Science, Cairo University, Giza 12613, Egypt

**Keywords:** probiotics, bifidobacteria, viability, heat-treated yeasts as nutrient, antioxidant capacity

## Abstract

The improvement of milk dairy products’ quality and nutritional value during shelf-life storage is the ultimate goal of many studies worldwide. Therefore, in the present study, prospective beneficial effects of adding two different industrial yeasts, *Kluyveromyces lactis* and *Saccharomyces cerevisiae* pretreated by heating at 85 °C for 10 min to be inactivated, before fermentation on some properties of ABT fermented milk were evaluated. The results of this study showed that the addition of 3% and 5% (*w*/*v*) heat-treated yeasts to the milk enhanced the growth of starter culture, *Lactobacillus acidophilus*, *Bifidobacteria,* and *Streptococcus thermophilus*, during the fermentation period as well as its viability after 20 days of cold storage at 5 ± 1 °C. Furthermore, levels of lactic and acetic acids were significantly increased from 120.45 ± 0.65 and 457.80 ± 0.70 µg/mL in the control without heat-treated yeast to 145.67 ± 0.77 and 488.32 ± 0.33 µg/mL with 5% supplementation of *Sacch. cerevisiae* respectively. Moreover, the addition of heat-treated yeasts to ABT fermented milk enhanced the antioxidant capacity by increasing the efficiency of free radical scavenging as well as the proteolytic activity. Taken together, these results suggest promising application of non-viable industrial yeasts as nutrients in the fermentation process of ABT milk to enhance the growth and viability of ABT starter cultures before and after a 20-day cold storage period by improving the fermented milk level of organic acids, antioxidant capacity, and proteolytic activities.

## 1. Introduction

In recent years, many studies have demonstrated the relation between the increased consumption of fermented and/or functional foods and a decrease in the predisposing of several diseases [1]. Functional foods have recently gained growing interest as healthy foods in many countries as they have a therapeutic effect on the health of consumers [1].

The modified functional foods include a wide range of ingredients, such as probiotics, prebiotics, vitamins, and minerals, which are found in different dairy products, such as fermented milks [2].

A probiotic microorganism is defined as a viable microbial dietary supplement to food that beneficially affects the host through its effects in the intestinal tract [3,4]. Two main genera of Gram-positive bacteria *Lactobacillus* and *Bifidobacterium* are used as probiotics [5]. The probiotics bacterium *Bifidobacteria animalis* subsp. *lactis* BB-12 and different strains of *Lactobacillus acidophilus* have many health beneficial effects toward the host, such as enhancement of the immune system [6], reduction of endotoxemia, attenuation of gut dysbiosis through the modulation of gut microbiota, reduction of plasma cholesterol levels [7], and antimicrobial, anticarcinogenic, and antioxidant activities [8]. Fermented milk combined with *Lactobacillus acidophilus* LA-5 and *Bifidobacterium* BB-12 represents a therapeutic agent for gastrointestinal disorders, such as bacteria/antibiotic-associated diarrhea [9]. In addition, probiotic yeast species, such as *Saccharomyces boulardii*, were reported to have the same therapeutic effects [10].

Dairy products are good vehicles for probiotics as well as prebiotics, a substance selectively utilized by host microorganisms, such as gut microbiota, stimulating beneficial host health effects [11]. Furthermore, dairy products represent a good environment for the growth of different probiotic strains [12]. The combination of two different starter cultures may enhance the fermentation process, for example, the co-culture of yoghurt with low acid production; *Lactobacillus casei*-01 and *Bifidobacterial* Bb-12 did not negatively affect the growth of both probiotic strains [13]. Different companies as suppliers for starter culture produce a wide range of probiotic cultures as direct vat set starter for milk fermentation. ABT mixed culture containing *Lactobacillus acidophilus*, *Bifidobacterial* sp., and *Streptococcus thermophilus* is produced by Danish Chr. Hansen Company and is applied as a probiotic culture in the Egyptian market.

The viability of different probiotic strains throughout the shelf life of dairy products as a carrier represents a challenge for many researchers [14,15,16]. The probiotic bacteria viability in fermented milks depends on a wide range of different factors, such as the strains used, interaction between starter cultures, culture conditions, chemical composition of milk and its final acidity, growth promoters and inhibitors, dissolved oxygen (especially for *Bifidobacterium* sp.), level of inoculation, and storage temperature [17,18]. Generally, *Lactobacillus* strains show good cellular stability throughout storage period. However, the cell counts of *Bifidobaterium lactis* LAFTI^®^ B94 are decreased by one log cycle at the end of the storage period [19].

On the other hand, some methods have been applied in order to enhance the viability of probiotics in fermented milks, such as microencapsulation [20], as the addition of viable *Saccharomyces boulardii* could enhance the viability and physicochemical properties of goat’s yoghurt and traditional yoghurt, respectively. This yeast acts as a source of nutrients and vitamins for bacterial starter culture, and it decreases the concentration of lactic acid released during storage [21,22]. However, major deviations were encountered in the end-product’s properties due to the supplemented viable yeasts, such as ethanol and gas production from fermented milk sugar (galactose and glucose residues) and yeasty flavor [21,23]. Therefore, in the present study, heat-treated yeasts were used during ABT milk fermentation to overcome the major constraints limiting the incorporation of viable yeasts by combining two inactivated industrial yeasts: *K. lactis* and *Sacch. cerevisiae* (heat pretreated) with different concentrations in order to improve the survivability of ABT starter cultures and the fermented milk organic acid profile. This study also examined the antioxidant and proteolytic activities of ABT milk after fermentation as well as a cold storage period of 20 days.

## 2. Materials and Methods

### 2.1. Materials

Fresh whole cows’ milk was obtained from a dairy unit, Faulty of Agriculture, Cairo University, Egypt. Skim milk powder was obtained from Arla Foods Co. (Stockholm, Sweden). Skim milk powder was used to standardize the total solids (TSs) of the cows’ milk to 14%.

Freeze-dried ABT-2 mixed starter culture containing *Lactobacillus* (Lb.) *acidophilus* LA-5, *Bifidobacterium* sp., and *Streptococcus thermophilus* was generously gifted by MIFAD Co., Cairo, Egypt. *Kluyveromyces* (K.) *lactis* NRRL Y-8279 was obtained from Northern Regional Research Laboratory (NRRL). *Saccharomyces cerevisiae* DSMZ 70 449 was obtained from the German collection of microorganisms and cell cultures GmbH (Braunschweig, Germany).

The microbiological media (de Man, Rogosa, Sharp (MRS) agar, M17 agar, malt extract broth) was obtained from Oxoid Ltd. (Basingstoke, Hampshire, UK). Anaerogen sachets were obtained from Oxoid Ltd. Vancomycin and L-cysteine hydrochloride were obtained from Merck Co. (Darmstadt, Germany).

### 2.2. Methods

#### 2.2.1. Preparation of Heat-Treated Yeast

Sterilized malt extract (ME) broth media was used to activate the lyophilized yeast strains prior to use and the cultures were incubated aerobically at 37 °C for 18 h then centrifuged at 3000× *g* for 5 min at 4 °C. Then, the pellets were harvested (~10^7^ cfu/g), washed twice, and re-suspended in normal saline and laboratory pasteurized at 85 °C for 10 min. The heat-treated cells were collected by centrifugation as mentioned above. In order to confirm the inactivation of the heat-treated cells, 100 µL of yeast suspension were examined by spreading onto ME agar medium. The heat-treated yeasts were used in the fermentation experiments when no visible growth was detected on the subculture ME agar medium after 24 h of incubation at 37 °C. Yeast suspension without heat treatment was used as a positive control, whereas the negative control was saline solution only.

#### 2.2.2. Production of Fermented Milk by ABT

Standardized cows’ milk (fat, 3.5%; protein, 3.8%; TS, 14%) was heated at 90 °C for 10 min then cooled to 40 °C and inoculated with 0.02% freeze-dried ABT-2 starter culture. The inoculated milk was divided into four equal portions, and each portion was inoculated with either 3% or 5% (*v*/*v*) heat-treated cells of *Sacch. cerevisiae* and *K. lactis*, respectively. The different treatments were dispended into 150-mL polystyrene cups and incubated at 40 °C until the pH reached 4.6. The viable count of ABT-2 starter culture was measured at the beginning and at the end of fermentation period. The fermented milks were stored for 20 days at 5 ± 1 °C.

#### 2.2.3. Chemical Analysis

Standardized cow’s milk and ABT fermented milk samples were analyzed for total solids (TSs), fat (Gerber method), protein (Kjeldahl method), and titratable acidity (TA, %) according to A. P. H. A. [24].

#### 2.2.4. Evaluation of the Viability of Starter Culture

The viability of ABT starter culture in different fermented milk treatments was measured after fermentation and the cold storage period. In order to count the viable cells of *Lb. acidophilus*, MRS agar medium at a lower pH value (5.5) was used to inhibit the growth of *St. thermophilus* [25]. MRS agar supplemented with 0.05% (*w*/*v*) L-cysteine hydrochloride and 0.3% (*w*/*v*) lithium chloride was used for counting the viable cells of *Bifidobactieria* sp. [26]. However, M17 agar was used for enumerating the viable count of *St. thermophilus* [27]. Plates of *Lb. acidophilus* and *St. thermophilus* were incubated aerobically at 43 °C for 48 h; however, plates of *Bifidobacteria* were incubated anaerobically at 37 °C for 72 h. The growth of ABT starter culture on the above-mentioned media was confirmed by Gram staining. The experiments were repeated three times under the same conditions, and all viable cell counts were performed in triplicates.

#### 2.2.5. Organic Acid Profile

The organic acid profiles of all ABT fermented milk samples were measured using high-performance liquid chromatography (HPLC) as previously described by Elshaghabee et al. [28]. Briefly, whey was collected from each sample by centrifugation at 6000× *g* for 20 min at 5 °C and the supernatant was filtered using a 0.45-µm membrane filter (Millipore Corp., Billerica, MA, USA). Samples were diluted with sulphuric acid 0.0085 N (1:25) on a Metacarb 87H column 7.5 × 300 mm (VWR Corporate Headquarters, Radnor, PA, USA). The mobile phase was sulphuric acid 0.0085 N with a flow rate of 0.3 mL/min. The Metacarb HPLC column was coupled with a reflective index (RI) detector and heated at a temperature of 65 °C.

#### 2.2.6. Antioxidant Activity

The antioxidant activity of different ABT fermented milk treatments was assessed as DPPH free radical scavenging activity [29].

After 30 min of reaction at room temperature, the sample absorbances at 517 nm (Jenway spectrophotometer 6300, Staffordshire, UK) were measured. The DPPH free radical scavenging activity was calculated as: % Radical scavenging percentage = [(A0 − A1)/A0] × 100, where A0 = the absorbance of blank and A1 = absorbance of test samples. Ascorbic acid was used as a positive control.

#### 2.2.7. Proteolytic Activity

Levels of protein hydrolysis of different ABT fermented milks treatment and control ABT without treatment were measured using the o-phthaldialdehyde (OPA) method as described by Donkor et al. [19]. The filtrate of each sample was incubated at room temperature with OPA for 4 min and the absorbance of the solution was measured at 340 nm as free amino groups by a spectrometer. The relative proteolytic activity was obtained upon comparison with unfermented milk substrates.

#### 2.2.8. Statistical Analysis

All obtained data in this study were analyzed using statistical methods by the Duncan test in the SPSS system 11.5 (SPSS, Inc.© Chicago, IL, USA). The results represent the mean ± standard deviations of triplicate independent experiments. Differences between means were considered significant at *p* < 0.05.

## 3. Results

**Figure 1:**
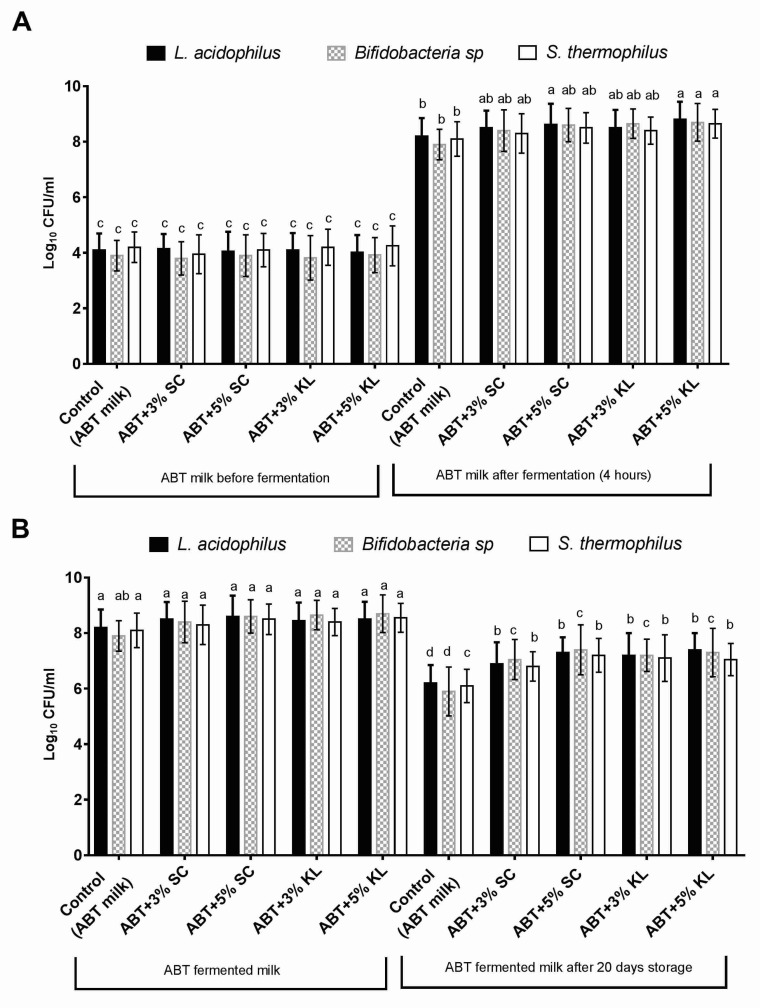
The viable counts (Log cfu/mL) of *Lb. acidophilus*, total *Bifidobacteria* sp., and *St. thermophilus* in control ABT milk before and after 4 h of fermentation (**A**) and before and after prolonged cold storage for 20 days (**B**). The bars on the graph represent mean ± SD of triplicate independent experiments (*n* = 3). The different letters above the bars show significant differences (*p* < 0.05) within the same bacterial starter culture species. Mean values sharing at least one common letter are not significantly different. Where ABT is the control; ABT+3% SC: ABT fermented milk containing 3% heat-treated *Saccharomyces cerevisiae* DSMZ 70 449 (*v*/*v*), ABT+5% SC: ABT fermented milk containing 5% heat-treated *Saccharomyces cerevisiae* DSMZ 70 449 (*v*/*v*), ABT+3% KL: ABT fermented milk containing 3% heat-treated *Kluyveromyces lactis* NRRL Y-8279 (*v*/*v*), ABT+5% KL: ABT fermented milk contains 5% heat-treated *Kluyveromyces lactis* NRRL Y-8279 (*v*/*v*).

In this study, the effect of the addition of heat-treated *K. lactis* and *Sacch. cerevisiae* on the growth behavior and viability of mixed ABT starter culture after milk fermentation was first evaluated. The results indicated that the mean values of viable counts of *Lb. acidophilus*, *Bifidobacterial* sp., and *St. thermophilus* were increased at the end of the fermentation period conducted at 40 °C for 4 h (Figure 1A). Indeed, the range of change in the viable counts for *Lb. acidophilus*, *Bifidobacterial*, and *St. thermophilus* was a 0.30–0.40, 0.50–0.70, and 0.20–0.40 increase in the log cycle, respectively, when heat-treated *Sacch. cerevisiae* was added at 3% and 5% (*v*/*v*) compared with untreated control ABT milk. On the other hand, this range reached 0.30–0.6, 0.75–0.80, and 0.30–0.55 log cycle for *Lb. acidophilus*, *Bifidobacterial*, and *St. thermophilus*, respectively, when *K. lactis* was added at 3% and 5% (*v*/*v*). However, it was noticed that only the heat-treated *K. lactis* at the 5% concentration formula significantly increased the levels of viable counts of all ABT starter cultures compared with the control under the same conditions. Nonetheless, heat-treated *Sacch. cerevisiae* at the 5% concentration significantly increased the levels of the viable counts of *Lb. acidophilus* only compared with the control. These results indicate a potential enhancement in the growth levels of ABT starter cultures as a result of the addition of *K. lactis* compared to *Sacch. cerevisiae* under the same conditions at the level of 5% (Figure 1A).

After 20 days of cold storage, the viable counts of each of the starter cultures, *Lb. acidophilus*, *bifidobacteria*, and *St. thermophilus*, were tested in fermented milks with or without supplemented heat-treated yeast. The results revealed that all starter culture viable counts in fermented milk supplemented with heat-treated *Sacch. cerevisiae* and *K. lactis* were increased significantly (*p* < 0.05) compared with control fermented milk under the same conditions within the same bacterial species (Figure 1B). The range of viable counts of *Lb. acidophilus*, *bifidobacteria*, and *St. thermophilus* were 6.90–7.40, 7.05–7.30, and 6.80–7.05 log cfu/g, respectively. Whereas, the levels of viable counts for *Lb. acidophilus*, *bifidobacteria*, and *St. thermophilus* were 6.20 ± 0.65, 5.90 ± 0.88, and 6.10 ± 0.60 log cfu/g, respectively (Figure 1B). Furthermore, the increase in the percentage of both supplemented heat-treated yeasts from 3% to 5% showed no significant change in the viable counts of ABT mixed starter culture (Figure 1B).

**Figure 2:**
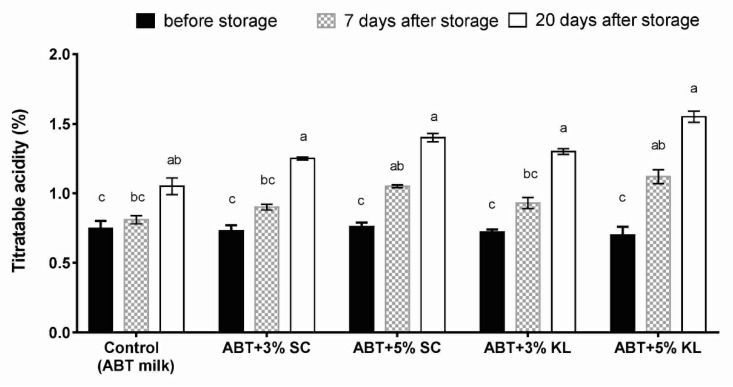
The changes of titratable acidity of different ABT fermented milk treatments during the prolonged cold storage period. The bars on the graph represent the mean values of titratable acidity percentage ± SD of triplicate independent experiments (*n* = 3). The different letters above the bars show significant differences (*p* < 0.05). Mean values sharing at least one common letter are not significantly different. Where ABT is the control without treatment; ABT+3% SC: ABT fermented milk contains 3% heat-treated *Saccharomyces cerevisiae* DSMZ 70 449 (*v*/*v*), ABT+5% SC: ABT fermented milk contains 5% heat-treated *Saccharomyces cerevisiae* DSMZ 70 449 (*v*/*v*), ABT+3% KL: ABT fermented milk containing 3% heat-treated *Kluyveromyces lactis* NRRL Y-8279 (*v*/*v*), ABT+5% KL: ABT fermented milk containing 5% heat-treated *Kluyveromyces lactis* NRRL Y-8279 (*v*/*v*).

Measurements of titratable acidity (%) after cold storage of fermented milk of each treatment formula demonstrated that the acidity levels in different ABT treatments as well as the ABT milk (control) were significantly increased after 20 days of cold storage. However, the titratable acidity was significantly higher after 7 days of treatment of ABT fermented milk with 5% supplementation of both *K. lactis* and *Sacch. cerevisiae* heat-treated yeasts but not with 3% compared with the ABT control (Figure 2).

**Figure 3:**
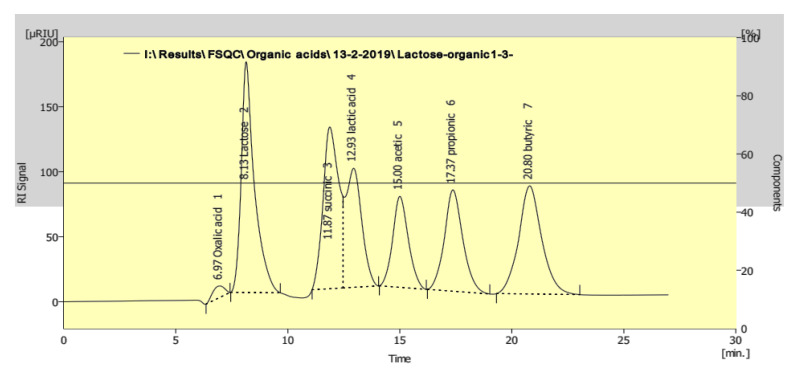
Chromatogramic diagram showing the separation of mixture standard (lactose and different organic acids) as analyzed by HPLC.

Therefore, the levels of organic acids in the fermented milk were measured by HPLC using a mixture of standard organic acids (Figure 3). The results showed modulation in the levels of organic acids (lactic and acetic acids) as a result of the addition of 3% and 5% of heat-treated yeasts after cold storage. The levels of lactic and acetic acids were significantly increased compared to the control ABT milk without heat-treated yeast after 20 days of cold storage (Table 1). Before storage (0 days), the level of acetic acid in different ABT fermented milk supplemented with heat-treated yeasts was higher than the ABT milk control. The maximum recorded lactic acid concentration was 145.67 ± 0.77 and 143.40 ± 1.05 µg/mL with 5% supplementation of both *Sacch. cerevisiae* and *K. lactis*, respectively, compared with the control without heat-treated yeast of 120.45 ± 0.65 µg/mL. In contrast, the levels of acetic acids in the different ABT milks supplemented with heat-treated yeasts were similar at the end of the cold storage period.

**Figure 4:**
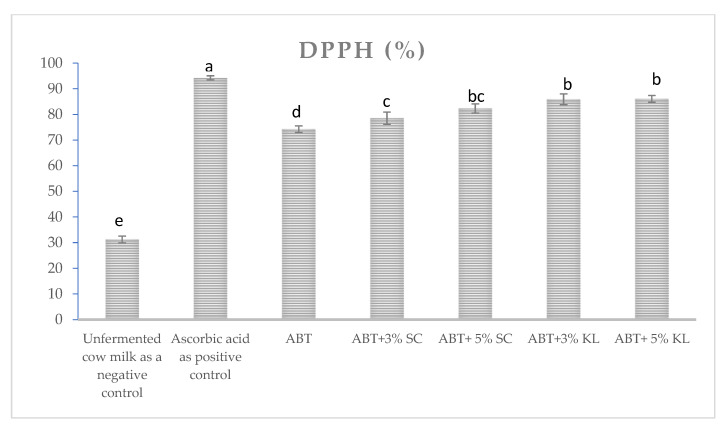
The DPPH scavenging activity of different ABT fermented milk with or without treatments. The bars on the graph represent the mean value of DPPH percentage ± SD of triplicate independent experiments (*n* = 3). The different letters above the bars show significant differences (*p* < 0.05).; Ascorbic acid is the positive control, ABT: control fermented milk without the addition of heat-treated yeasts, ABT+3% SC: ABT fermented milk containing 3% heat-treated *Saccharomyces cerevisiae* DSMZ 70 449 (*v*/*v*), ABT+5% SC: ABT fermented milk containing 5% heat-treated *Saccharomyces cerevisiae* DSMZ 70 449 (*v*/*v*), ABT+3% KL: ABT fermented milk containing 3% heat-treated *Kluyveromyces lactis* NRRL Y-8279 (*v*/*v*), ABT+5% KL: ABT fermented milk containing 5% heat-treated *Kluyveromyces lactis* NRRL Y-8279 (*v*/*v*).

The proteolytic activities during the cold storage period were significantly increased after 7 and 20 days as shown in Table 2. Before storage, no significant change in proteolytic activities could be detected with all treatment of 3% heat-treated yeasts. On the contrary, there was a significant increase in the formula with 5% heat-treated *K. lactis* compared with ABT milk without treatment. The increase in proteolytic activities was more pronounced after 20 days of cold storage, with a significant difference between 3% and 5% supplementation of heat-treated *K. lactis*. On the other hand, DPPH free radical scavenging activity was tested as an indicator of antioxidant capacity in ABT milk before and after fermentation as well as ABT fermented milk supplemented with 3% and 5% heat-treated yeast. The results revealed a significant increase in the percentage of DPPH scavenging activities and antioxidant capacity in both heat-treated yeasts compared to control ABT fermented milk (Figure 4).

## 4. Discussion

The present study shows a correlation between the addition of heat-treated, in other words non-viable, yeast and the enhancement of the growth and viability of probiotic starter culture (ABT) during the fermentation period as well as during 20 days of cold storage as shown in the values of viable counts of *Lb. acidophilus*, *Bifidobacteria* sp., and *St. thermophilus*. The tested heat-treated *Sacch. cerevisiae* increased the viable count of *Lb. acidophilus* at the 5% concentration whereas *K. lactis* improved the growth of all starter cultures but only at the 5% addition. This is due to the fastidious nature of lactic acid bacteria (LAB) and *bifidobacteria* in terms of its nutritional requirements for growth and performance [30]. The nutritional potential of the surrounding environment, such as free amino acids, short peptides, as well as oligosaccharides, affects the growth and viability of fermented milk starter cultures [31]. The heat-treated yeast provides a critical fermentation media ingredient for the growth of ABT starter culture that supports the strain-dependent nutritional needs and their concentrations to maintain a viable cell count.

Some studies reported a positive effect of the addition of different species of *Saccharomyces* on the growth of probiotic bacteria during the fermentation process of milk. It was reported that the viability of *Bifidobacteria longum* in milk can be improved by the addition of 0.01% *Sacch. cerevisiae* [32]. Furthermore, *Sacch. boulardii* induces the growth and survival of LAB during the milk fermentation process [33]. However, scarce or no studies have reported the use of non-viable yeast in the fermentation media.

In order to maintain a proper probiotic cell count during cold storage of fermented milk, milk different dietary supplement formats and prebiotics have been used to ensure the stability of probiotics [31,34]. In this regard, it was reported that the addition of oligofructose and polydextrose, to formulate the fermented milk in such a way that promotes the probiotic bacteria viability, such as *Lb. rhamnosus*, *Lb. acidophilus*, and *Bifidobacterium* spp., during cold storage, was effective [34]. Alternatively, the micro-encapsulation technology was used to protect probiotic strains that are sensitive during cold storage from the fermented milk matrix until the moment of consumption [20]. In our study, the best formula for the ABT fermented milk preparation was the addition of 5% heat-treated *K. lactis* as it not only stimulated the ABT starter culture counts during fermentation but also after 20 days of cold storage.

Previous studies reported that the fortification of camel milk, fermented by ABT-type culture, with honey (black locust) at 5% improved retention of the viability of *Bifidobacteria* during refrigerated storage up to 35 days at 4 °C. This finding was hypothesized by the hone’s various oligosaccharide contents, with a low degree of polymerization, which are favored substrates for *Bifidobacterial* support [35].

In order to obtain a high-performance ABT fermented milk end-product, the cells’ ability to survive and adjust to the stresses imposed by the manufacturing process is a key factor. The limited viability of LAB because of their susceptibility to low pH can be reversed by the ability of *Sacch. boulardii* to metabolize organic acids. In commercial probiotic fermented milks, many studies have demonstrated that different probiotics, such as *Lactobacilli* and *Bifidobacteria*, show a significant decline in their viability during the product’s shelf life [13,36,37]. Therefore, in our study, heat-treated *K. lactis* or *Sacch. cerevisiae* were supplemented to ABT milk in order to enhance the viability of *Lb. acidophilus*, *Bifidobacteria* sp., and *St. thermophilus* in fermented milk throughout its commercial life because they might act as a source of nutrients, e.g., nitrogen source and various oligosaccharides, which are essential for the growth and viability of these microorganisms [38]. Furthermore, this combination with health-promoting ABT starter culture might improve the therapeutic effects as well as their viability over the storage period of the products. Several methods were reported to enhance the fermentation profile of milk by probiotics, such as the addition of stimulation compounds, enzymatic catalysis, and irradiation with red laser and ultrasound [38,39,40].

It was observed that in all heat-treated yeast formulas, no statistically significant changes in the titratable acidity and lactic acid concentrations after 4 h of fermentation compared with the ABT fermented milk control were recorded. These results suggest that the tested formula decelerates the post-acidification and lactic acid release. The same observations were reported after addition of prebiotics compounds, oligofructose and polydextrose, which led to lower post-acidification and lactic acid release [34].

Because of the enhancement in the viability of ABT starter culture, levels of acidity and organic acids (lactic acid and acetic acid) were increased at the end of the 20 days of the cold storage period as analyzed by HPLC and titratable acidity. Indeed, the addition of viable *Sacch. boulardii* to yoghurt samples resulted in enhanced levels of acidity and the survival of yoghurt culture during cold storage [21,22].

The proteolytic activities revealed that no significant change could be detected with all treatments of 3% and 5% heat-treated yeasts before storage, but only the 5% heat-treated *K. lactis* formula increased the proteolytic activities, which follows the increase in the viable count of all ABT starter culture. This result suggests that this formula may provide a higher free amino acid than other formulas, which agrees with the findings that the initial high free amino acid content of milk increases the proteolytic activities of starter cultures during the fermentation process [41].

Furthermore, an enhancement of the levels of proteolytic activity was observed when 5% heat-treated yeasts were added to ABT milks after 20 days of cold storage. This could be attributed to the presence of more peptides from degradation of the heat-treated yeast cell wall into polysaccharides and protein [42]. The increase in proteolytic activities agrees with the results obtained by Niamah et al. [22], who showed that the addition of *Sacch. boulardii* to traditional yoghurt resulted in enhanced proteolytic activity of yoghurt samples; however, in this study, non-viable *Sacch. cerevisiae* and *K. lactis* were used.

The bacterial enzymatic activities during milk fermentation lead to the production of various biologically active peptides, by peptic digestion of milk protein, which have several potential health benefits. Some of these bioactive peptides represent naturally occurring antioxidants. The antioxidant activities of all formulated milk with heat-treated milk were reported to have higher antioxidant capacities than the tested commercial starter culture of ABT [43]. It is likely that the proteins and polymers from heat-treated yeast were metabolized by ABT cultures, leading to an increase in the concentration of antioxidant peptides and/or compounds after fermentation [44].

## 5. Conclusions

This is the first study that shows the application of non-viable yeast as a potential prospective supplement in the manufacture of fermented ABT milk. The best tested formula was heat-treated *K. lactis* at a concentration of 5% as it not only promotes the growth of ABT starter culture but also enhances the viability of *Lb. acidophilus* and *Bifidobacteria* as probiotics after the cold storage period (20 days, 5 ± 1 °C). Furthermore, modulation of the organic acid profile as well as the increased proteolytic and antioxidant activities were observed after fermentation and during cold storage up to 20 days in comparison to the control without supplement. Fortification of cultured dairy products with heat-treated yeast might be used as a prospective nutraceutical product.

## Figures and Tables

**Table 1:** Mean values of lactic and acetic acids (µg/mL) of control unfermented milk and ABT fermented milk without or with treatments after 20 days of cold storage.

Treatments *	Storage Period, Days	Concentration of Organic Acids (µg/mL)	
		Lactic Acid	Acetic Acid
Control Unfermented Milk	0	ND	ND
	15	ND	ND
ABT	0	75.25 ± 1.30 ^d,^**	285.60 ± 0.80 ^d^
	20	120.45 ± 0.65 ^c^	457.80 ± 0.70 ^b^
ABT+3% SC	0	85.30 ± 0.45 ^d^	290.78 ± 0.60 ^c^
	20	135.40 ± 0.85 ^b^	479.60 ± 0.85 ^a^
ABT+5% SC	0	86.35 ± 0.58 ^d^	295.85 ± 0.25 ^c^
	20	145.67 ± 0.77 ^a^	488.32 ± 0.33 ^a^
ABT+3% KL	0	85.35 ± 1.20 ^d^	293.25 ± 0.72 ^c^
	20	138.30 ± 0.52 ^b^	475.05 ± 1.06 ^a^
ABT+5% KL	0	88.45 ± 1.33 ^d^	289.90 ± 0.38 ^c^
	20	143.40 ± 1.05 ^a^	482.85 ± 1.25 ^a^

* ABT: ABT fermented milk without the addition of heat-treated yeasts, ABT+3% SC: ABT fermented milk containing 3% heat-treated *Saccharomyces cerevisiae* DSMZ 70 449 (*v*/*v*), ABT+5% SC: ABT fermented milk containing 5% heat-treated *Saccharomyces cerevisiae* DSMZ 70 449 (*v*/*v*), ABT+3% KL: ABT fermented milk containing 3% heat-treated *Kluyveromyces lactis* NRRL Y-8279 (*v*/*v*), ABT+5% KL: ABT fermented milk containing 5% heat-treated *Kluyveromyces lactis* NRRL Y-8279 (*v*/*v*). data mean ±: Standard deviation. ** The different letters represent significant differences (*p* < 0.05) in each group. Mean values sharing at least one common letter are not significantly different.

**Table 2:** Changes in the relative proteolytic activity (as % degree protein hydrolysis) before storage and after 7 and 20 days of cold storage of control ABT fermented milk and different treatments of ABT milk.

Treatments	Before Storage	After 7 Days	After 20 Days Cold Storage
ABT	3.15 ± 0.07 ^f,^**	3.50 ± 0.07 ^e^	3.84 ± 0.12 ^c,d^
ABT + 3% SC	3.31 ± 0.11 ^f^	3.68 ± 0.06 ^d,e^	3.92 ± 0.04 ^b,c^
ABT + 5% SC	3.51 ± 0.05 ^f^	3.72 ± 0.03 ^d,e^	4.21 ± 0.15 ^b^
ABT + 3% KL	3.42 ± 0.20 ^f,g^	3.81 ± 0.15 ^c,d^	4.13 ± 0.23 ^b^
ABT + 5% KL	3.71 ± 0.06 ^d,e^	3.92 ± 0.25 ^b,c^	4.45 ± 0.13 ^a^

ABT: control; ABT fermented milk without addition of heat-treated yeasts, ABT+3% SC: ABT fermented milk containing 3% heat-treated *Saccharomyces cerevisiae* DSMZ 70 449 (*v*/*v*), ABT+5% SC: ABT fermented milk contains 5% heat-treated *Saccharomyces cerevisiae* DSMZ 70 449 (*v*/*v*), ABT+3% KL: ABT fermented milk containing 3% heat-treated *Kluyveromyces lactis* NRRL Y-8279 (*v*/*v*), ABT+5% KL: ABT fermented milk containing 5% heat-treated *Kluyveromyces lactis* NRRL Y-8279 (*v*/*v*). Data mean value ± standard deviation. ** The different letters represent significant differences (*p* < 0.05) in each group. Mean values sharing at least one common letter are not significantly different.

## Data Availability

Data is contained within the article.

## Abbreviations

ABT	*acidophilus-bifidus-thermophilus*
NRRL	Northern Regional Research Laboratory
MRS	de Man, Rogosa, Sharp
ME	malt extract
TA	titratable acidity
HPLC	high-performance liquid chromatography
DPPH	Diphenylpicrylhydrazyl
OPA	o-phthaldialdehyde
CFU	colony forming unit
LAB	lactic acid bacteria
